# Tandem mass spectrometry data quality assessment by self-convolution

**DOI:** 10.1186/1471-2105-8-352

**Published:** 2007-09-20

**Authors:** Keng Wah Choo, Wai Mun Tham

**Affiliations:** 1 Bioinformatics Group, Nanyang Polytechnic, 569830 Singapore, Republic Of Singapore

## Abstract

**Background:**

Many algorithms have been developed for deciphering the tandem mass spectrometry (MS) data sets. They can be essentially clustered into two classes. The first performs searches on theoretical mass spectrum database, while the second based itself on *de novo *sequencing from raw mass spectrometry data. It was noted that the quality of mass spectra affects significantly the protein identification processes in both instances. This prompted the authors to explore ways to measure the quality of MS data sets before subjecting them to the protein identification algorithms, thus allowing for more meaningful searches and increased confidence level of proteins identified.

**Results:**

The proposed method measures the qualities of MS data sets based on the symmetric property of b- and y-ion peaks present in a MS spectrum. Self-convolution on MS data and its time-reversal copy was employed. Due to the symmetric nature of b-ions and y-ions peaks, the self-convolution result of a good spectrum would produce a highest mid point intensity peak. To reduce processing time, self-convolution was achieved using Fast Fourier Transform and its inverse transform, followed by the removal of the "DC" (Direct Current) component and the normalisation of the data set. The quality score was defined as the ratio of the intensity at the mid point to the remaining peaks of the convolution result. The method was validated using both theoretical mass spectra, with various permutations, and several real MS data sets. The results were encouraging, revealing a high percentage of positive prediction rates for spectra with good quality scores.

**Conclusion:**

We have demonstrated in this work a method for determining the quality of tandem MS data set. By pre-determining the quality of tandem MS data before subjecting them to protein identification algorithms, spurious protein predictions due to poor tandem MS data are avoided, giving scientists greater confidence in the predicted results. We conclude that the algorithm performs well and could potentially be used as a pre-processing for all mass spectrometry based protein identification tools.

## Background

### Mass spectrometry

Mass spectrometry (MS) is a common analytical technique used to identify unknown compounds, quantify known materials, and elucidate the molecular structure and chemical composition of organic and inorganic substances. A mass spectrometer is an instrument used to measure the mass-to-charge ratio of individual molecules that have been converted into electrically charged molecules, or ions [1]. These ions are filtered and ordered from a lower to higher mass-to-charge ratio (m/z) before passing through an ion detector in the instrument [2]. In the field of proteomic analysis, matrix assisted laser desorption ionisation (MALDI) and electrospray ionization (ESI) are two ionisation techniques generally used. Mass spectrometry is currently experiencing rapid growth in mass-spectrometry-based biomarker discovery and clinical proteomics, where hundreds of proteins can be sequenced quickly. As a consequence, large amounts of proteomics data are produced and made available to the public [3-5].

Although the generation of raw MS spectra has become easier, the analysis and identification of the data still post many challenges. Many protein identification tools have been developed, such as PEAKS [6] MASCOT [7,8], Phenyx [9], SEQUEST [10] and OMSSA [11]. In the case of high throughput proteomics, it involves the analysis of hundreds of thousands of peptide spectra derived from biological samples. Four general types of algorithms can identify these spectra,

*1. De novo *calling of the sequence directly from the spectrum [6,12,13].

2. Use of unambiguous "peptide sequence tags" derived from spectra that are used to search known sequences [14-16].

3. Cross-correlation methods that correlate experimental spectra with theoretical spectra [17,18].

4. Probability-based matching that calculates a score based on the statistical significance of a match between an observed peptide fragment and those calculated from a sequence search library [7,19-22].

Cross-correlation methods and probability-based matching are two well-received methods for protein identification. In these methods, a theoretical mass spectra database is first generated from known protein sequences. To search this database with experimental spectra, the correlation of the experimental and theoretical spectra is calculated. Based on the statistical properties of the protein database and the correlation values (actual implementation is more complex), a score is given for the matched spectra.

Most of these tools have attained a certain degree of success thus far; nevertheless reliable protein identification using these methods is still a time-consuming and program-dependent task. A considerable frequency of false positive protein identifications has been reported from independent studies [23,24]. Knowing that the quality of mass spectra is crucial in protein identification, several attempts to address the issue have been made using some information obtained from mass spectra generated by fragmented peptides [25-28]. In particular, Purvine *et al *[27] used a prefilter with three features for tandem MS spectra classification; one feature addressed the uncertainty in charge state assignments, the second was based on total signal intensity and the third on a signal-to-noise estimate. They obtained good results by adjusting these features. Although these approaches have been useful, we introduce an additional prefilter feature based on the symmetry property of the b- and y-ions, to compliment and improve the pre-filter process.

### Convolution

Convolution is a mathematical operation commonly used in digital signal processing (DSP). For discrete time series, the convolution is given as:

hi=∑j=0mfigi−j
 MathType@MTEF@5@5@+=feaafiart1ev1aaatCvAUfKttLearuWrP9MDH5MBPbIqV92AaeXatLxBI9gBaebbnrfifHhDYfgasaacH8akY=wiFfYdH8Gipec8Eeeu0xXdbba9frFj0=OqFfea0dXdd9vqai=hGuQ8kuc9pgc9s8qqaq=dirpe0xb9q8qiLsFr0=vr0=vr0dc8meaabaqaciaacaGaaeqabaqabeGadaaakeaacqWGObaAdaWgaaWcbaGaemyAaKgabeaakiabg2da9maaqahabaGaemOzay2aaSbaaSqaaiabdMgaPbqabaGccqWGNbWzdaWgaaWcbaGaemyAaKMaeyOeI0IaemOAaOgabeaaaeaacqWGQbGAcqGH9aqpcqaIWaamaeaacqWGTbqBa0GaeyyeIuoaaaa@3F95@

where *f*_*j *_and *g*_*j *_are two time series data sets. Self-convolution refers to convolution applied onto the same data series, where *g*_*i*-*j *_is the time-reversal copy of the data series *f*_*j*._

Self-convolution has been used in many applications, where symmetry property is key feature of the signal, such as those found in the field of digital communication [29] and image processing [30]. We will show in this work that MS do have such property inherited naturally from the fragmentation process, and hence the same approach can be used to extract information from the spectra. The success of this method depends on the availability of the complementary b- and y-ions, which are the two types of most commonly found ions in the conventional tandem mass spectrometry.

### Peptide fragmentation

Peptide fragmentation is a process where peptide fragment ions are generated by dissociation in an ion trap of a mass spectrometer. In this process, the breakage can occur between any bonds in the peptide, but commonly occurs at the peptide bond. When a peptide is fragmented at a single peptide bond between the carbonyl and nitrogen, two fragments are formed. In the case where one peptide fragment retains the positive charge at the C-terminus of the peptide ion, it is called a y-ion. If the fragment retains the positive charge at the N-terminus, it is known as a b-ion. When a singly charged peptide is fragmented, the charge is retained only at one terminus and only the fragment containing the charge is detected while the other fragment is lost as a neutral fragment. Doubly charged peptides tend to produce two singly charged ions, though sometimes doubly charged ions can also be formed.

**Figure 1:**
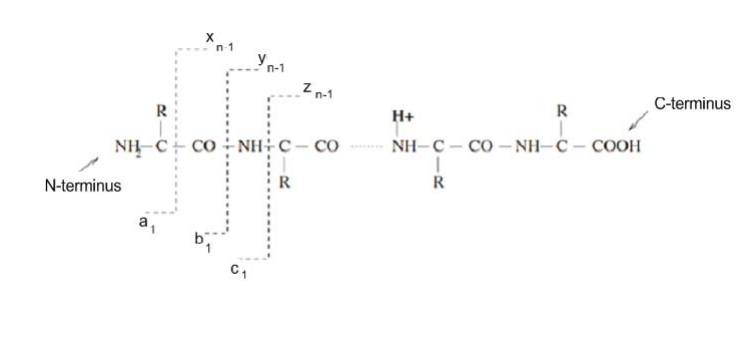
**Peptide fragmentation**. This figure shows various breakage points along a peptide bond and ions are formed in complementary to the N-terminal and C-terminal.

The types of fragment ions observed in an tandem MS spectrum depend on many factors, including primary peptide sequence, amount of internal energy and how the energy was introduced, charge state, etc. The accepted nomenclature for fragment ions was first proposed by Roepstorff and Fohlman [31], and subsequently modified by Johnson *et al *[32] and Biemann [33,34]. There are different dissociation methods available, including commonly used gas phase collision-induced dissociation (CID) [33], surface-induced dissociation [35], photodissociation [36], electron-capture dissociation [37], and electron transfer dissociation [38]. The b-ions and y-ions are usually formed when fragmentation occurs under low energy conditions. Fig. 1 shows all possible breakage points along a peptide bond.

**Figure 2:**
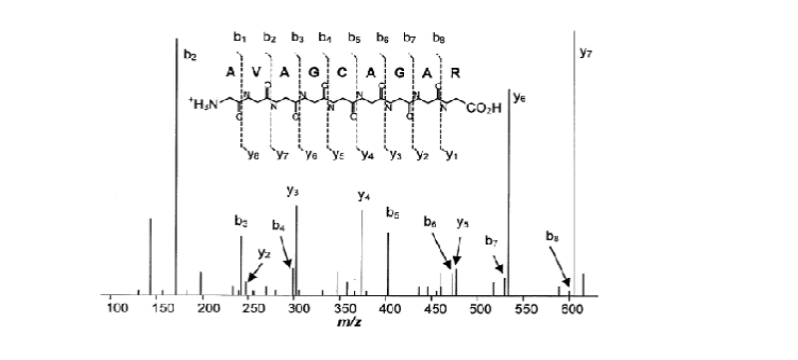
**Tandem mass spectrum**. This figure shows the possible fragmentation on the short peptide AVAGCAGAR and its respective intensity versus m/z mass spectrometry plot.

Other ions like a-ions and x-ions, which form a complementary pair, and c-ions and z-ions, which form another complementary pair, are also formed. The a-ions and x-ions are formed when the peptide fragments between the amino acid side chain and the carbonyl molecule. The c-ions and z-ions are formed when the peptide fragments between the nitrogen and the amino acid side chain molecule. These ions are formed when fragmentation occurs high-energy conditions since higher amounts of energy are required to break these bonds. Fig. 2 shows a typical tandem MS spectrum.

The development of chemical theory of peptide fragmentation [39,40] has enabled the de novo prediction of fragmentation spectra from peptide sequences. Using a kinetic model, Zhang made the first successful attempt at predicting the low-energy CID spectra of singly and doubly charged peptides [41]. Elias *et al*. [42] were first to successfully utilize a set of well-annotated fragmentation spectra acquired from an electrospray ion-trap mass spectrometer in an attempt to infer the probabilistic rules of fragmentation. More recently, Randy *et al*. used machine-learning algorithm to predict various fragment-ion types of doubly and triply charged precursor ions by learning peptide fragmentation rules in mass spectrometry in the form of posterior probabilities [43]. Yu *et al*. proposed a novel method to automatically learn the factors influencing fragmentation from a training set of tandem MS spectra [44]. Despite the availability of the various prediction models, it is unclear how these models could be used for predicting fragment ions in different types of mass spectrometry machines.

## Results

To validate the proposed method of tandem MS spectra assessment, we conducted series of tests on theoretical MS spectra as well as experimental MS spectra. The results of the tests on theoretical MS spectra are tabulated in Table 1. We then used another 60 sets experimental tandem MS spectra to tests its effectiveness and robustness.

**Table 1:** Scoring of theoretical mass spectrum under different conditions

**Protein Sequence: MTDQEAIQDLWQWR**					
**S/No**	**Descriptions**	**Mid-point m/z**	**Mid-point peak value**	**Average of 20 peaks**	**Quality Score**

**Test Section A**					
1	White Gaussian noise level = 0%	910.92	1.00	0.2149	4.6542
2	White Gaussian noise level = 5%	910.92	1.00	0.2195	4.5564
3	White Gaussian noise level = 10%	910.92	1.00	0.2162	4.6255
4	White Gaussian noise level = 15%	910.92	1.00	0.2820	3.5467
5	White Gaussian noise level = 20%	910.91	1.00	0.5616	1.7808
6	White Gaussian noise level = 25%	910.92	1.00	0.7875	1.2699
7	White Gaussian noise level = 30%	910.92	1.00	0.8570	0.9140
**Test Section B**					
1	add 10 random peaks, noise level 1	910.92	1.00	0.2150	4.6511
2	add 20 random peaks, noise level 1	910.92	1.00	0.2153	4.6442
**Test Section C**					
1	b-ions peaks reduced by 10%, noise level 1	910.92	1.00	0.2206	4.5330
2	b-ions peaks reduced by 20%, noise level 1	910.92	1.00	0.2263	4.4192
3	b-ions peaks reduced by 30%, noise level 1	910.92	1.00	0.2360	4.2380
4	b-ions peaks reduced by 40%, noise level 1	910.92	1.00	0.2510	3.9842
5	b-ions peaks reduced by 50%, noise level 1	910.92	1.00	0.2727	3.6673
6	b-ions peaks reduced by 60%, noise level 1	910.92	1.00	0.3335	2.9989
7	b-ions peaks reduced by 70%, noise level 1	910.92	1.00	0.4363	2.2654
8	b-ions peaks reduced by 80%, noise level 1	NA	-	-	-
**Test Section D**					
1	y-ions peaks reduced by 10%, noise level 1	910.92	1.00	0.2169	4.6106
2	y-ions peaks reduced by 20%, noise level 1	910.92	1.00	0.2198	4.5489
3	y-ions peaks reduced by 30%, noise level 1	910.92	1.00	0.2235	4.4740
4	y-ions peaks reduced by 40%, noise level 1	910.92	1.00	0.2303	4.3418
5	y-ions peaks reduced by 50%, noise level 1	910.92	1.00	0.2435	4.1072
6	y-ions peaks reduced by 60%, noise level 1	910.92	1.00	0.2956	3.3824
7	y-ions peaks reduced by 70%, noise level 1	910.92	1.00	0.3926	2.5468
8	y-ions peaks reduced by 80%, noise level 1	NA	-	-	-
**Test Section E**					
1	minus 2 b-ions peaks, noise level 1	910.92	1.00	0.2097	4.7692
2	minus 4 b-ions peaks, noise level 1	910.92	1.00	0.2320	4.3103
3	minus 6 b-ions peaks, noise level 1	910.92	1.00	0.3013	3.3190
4	minus 8 b-ions peaks, noise level 1	910.92	1.00	0.3435	2.9114
5	minus 10 b-ions peaks, noise level 1	NA	-	-	-
**Test Section F**					
1	minus 2 y-ions peaks, noise level 1	910.92	1.00	0.2512	3.9813
2	minus 4 y-ions peaks, noise level 1	910.92	1.00	0.3027	3.3041
3	minus 6 y-ions peaks, noise level 1	910.92	1.00	0.3810	2.6245
4	minus 8 y-ions peaks, noise level 1	910.92	1.00	0.4432	2.2562
5	minus 10 y-ions peaks, noise level 1	NA	-	-	-

In our work, we tested the qualitative measurement of the tandem mass spectra based on different noise intensities (Sec. A), additional spurious peaks (Sec. B), different b-ion intensities (Sec. C), different y-ion intensities (Sec. D), different percentage loss of b-ion (Sec. E), and different percentage loss of y-ion (Sec. F). We observed the drop in score as the quality of the theoretical mass spectrum deteriorates.

### Quantitative measurement of theoretical tandem MS spectra

We first compute the quality score (QS) on theoretical MS spectra based on our derivation shown in Eq. 1. The protein sequence [MTDQEAIQDLWQWR] was chosen arbitrary to form the theoretical spectra for our work. The theoretical spectra are subjected to different degradation processes, including introduction of white Gaussian noise, reduction in ion peak intensities, removal of ion peaks, as describe in the Method section. The test results are tabulated in Table 1.

In the first test, we included all the theoretical b and y-ions peaks in the spectrum, with white Gaussian noise (noise with normal distribution) of different amplitudes added. The scores are captured in Section A of Table 1. We observed that the QS scores remain stable for noise amplitudes between 0 and 10% of the peak intensity.

In the second test, we added in random peaks of equal amplitude to the b and y-ions in addition to the white Gaussian noise. The random peaks could represent spurious ion peaks intended to degrade the quality of the spectrum. We observed that with 10 and 20 random peaks added, the scores are not much affected, with QS equal to 4.6511 and 4.6442 respectively. This shows that the scores are not much affected by the random peaks, as long as the b and y-ions are intact.

**Figure 3:**
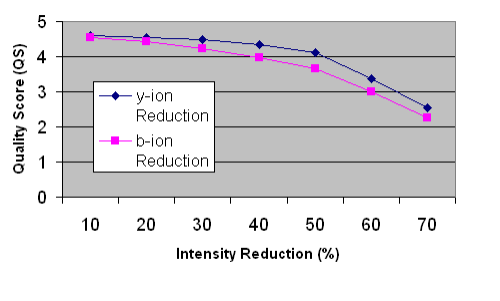
**Plot of QS versus ion intensity reduction**. This figure shows the effect of reduction in ion intensity on the QS score.

In the next two test scenarios, we reduced the intensity of b and y-ions to simulate the lack of fragmented b and y-ions in the spectrum. As b-ions reduce in intensity, the QS drops from 4.5330 to 2.2654 at 10% to 70% reduction of the b-ion intensity, as shown in Section C in Table 1. The reduction of y-ion intensity shows similar effect on the QS score, it drops from 4.6106 to 0.5468 at 10% to 70% reduction in intensity, as shown in Section E in Table 1. The results are shown in Fig. 3. As the intensity is reduced further, there is no longer any peak detected at the mid-point window of the self-convolution result.

Lastly, we removed randomly some of the b or y-ion peaks to simulate loss of certain ion fragments. The number of ions removed varies from 2 to 8 and we observed that the QS drop from 4.7692 to 2.9114 and from 3.9813 to 2.2562 for b-ion and y-ion loss respectively, as shown in Section E and Section F of Table 1. As the number of ion peak is further reduced, the mid-point peak is no longer detectable. These tests show the relation between the qualities of the spectrum to the QS that we established to assess the quality of the MS.

### Qualitative measurement of experimental tandem MS spectra

**Figure 4:**
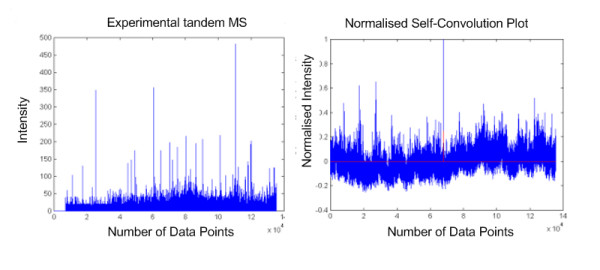
**Plot of self-convolution of experimental mass spectrum**. This figure shows the actual mass spectrum (left) and its respective self-convolution result (right). A high mid-point intensity might not indicate a good quality spectrum as a product of two high intensity peaks could generate it by chance.

We started the quality assessment by simply performing a self-convolution on some of the experimental MS spectra. Fig. 4 shows a plot of the result of self-convolution of one of the raw tandem MS spectra. Although the plot does show a high peak at the mid-point window of the result, we found out that the product of two high intensity peaks happened incidentally to be at the mid-point. This could cause misinterpretation and therefore erroneous for us to consider this result as an indication of good quality spectrum. We have thus further improved on the approach by considering side peaks and normalisation process.

**Figure 5:**
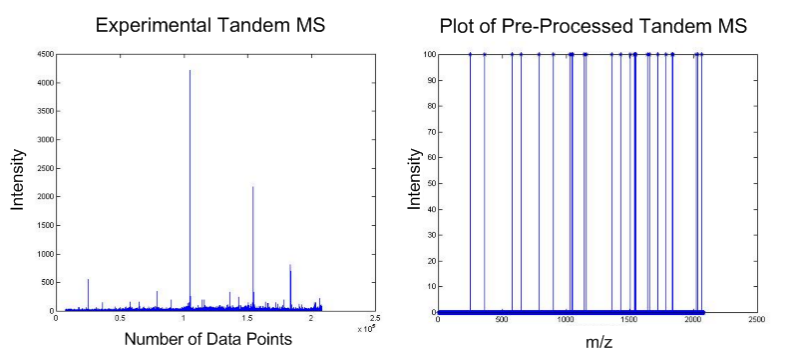
**Pre-processing of ion peaks intensities**. This figure shows a plot of the experimental tandem MS (left) and the newly generated mass spectrum after being pre-processed (right).

**Figure 6:**
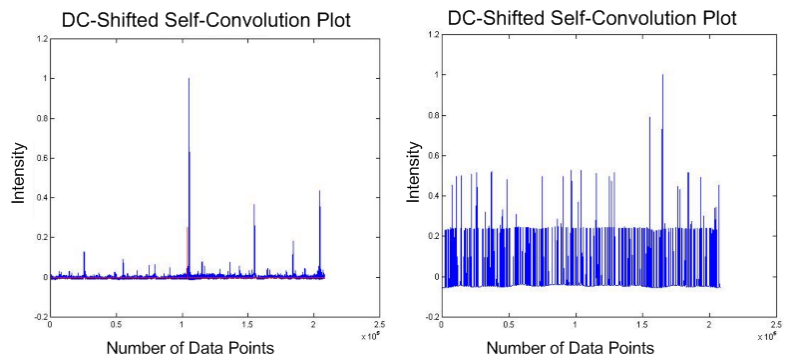
**DC-shifted self-convolution plot of experimental tandem MS**. This figure shows the difference between the DC-shifted self-convolution results obtained from the original mass spectrum (left) and the pre-processed mass spectrum (right).

The proposed method was subsequently tested on 60 sets of real tandem MS spectra (unpublished). They were subjected to the QS scoring function described in the Eq. 1. We considered 15 highest intensity peaks to the left and right of the mid-point window of each spectrum. The self-convolution result is shown in Fig. 5. The DC shifted self-convolution plots of the original tandem MS spectrum is contrasted with that of the newly generated plot, as shown in Fig. 6. We have also assumed that 30 peaks are sufficient in our calculation, but this number can be increased in the case where more ion fragments are expected. All tandem mass spectra having high scores have been identified successfully using MASCOT [8] with high confidence (> 45).

## Discussion

The fragmentation of peptide sequence using conventional mass spectrometer produces spectra consists mostly of b and y-ion peaks. The quality of the mass spectra depends therefore mainly on the presence of the b- and the y-ions in the spectra. Current state-of-the-art database search tools depend heavily on these ion peaks and the lack of such peaks would lead to no protein match, or in the worst case, the erroneous matching of proteins in the database. Some database search algorithms allow inclusion of a- and/or z-ions; such inclusion makes the search more complex and computationally intensive, hence significantly slows down the protein identification process.

We proposed a novel method where the quality of the mass spectrum is determined from self-convolution of the mass spectra. This approach complements existing methods in selecting good quality tandem MS spectra to be processed by database search and/or *de novo *sequencing. This method is unique, as it does not depend on the charge of the fragmented ion, nor its length. Random peaks such as those produced by machine noise or contaminants (*e.g*. Keratin), irregardless of its intensity will not affect the process, as it requires a complementary pair to work.

Knowing that the presence of a fair amount of complementary b- and y-ions constitute to good quality mass spectrum, we can be assured that by selecting spectra with high QS values, only good quality tandem MS are pre-filtered to be processed for protein identification.

We note that tandem MS spectra having non-complementary b and y-ions might score poorly using this approach. Examples of such spectra are those having large number of y-ions but only very few complementary b-ions, and vice versa.

## Conclusion

We conclude that the new approach is effective and useful in assessing the quality of tandem mass spectrum by analysing the self-convolution result of the spectra. This method relies mainly on the symmetry property inherited from the formation of complementary b and y-ions found in the tandem MS spectra. The proposed assessment scheme can be used to complement existing pre-filter/assessment processes to ensure that only good quality spectra are sent for protein identification process, reducing false positive protein detection by database search and *de novo *sequencing protein identification tools. This method can be further improved by taking into consideration of other complementary ions, such as a-ions and x-ions.

## Methods

We proposed a method that exploits the naturally inherited symmetry property of tandem mass spectrum. The symmetry property of the spectra formed by the combination of b- and y-ions can be observed easily from the spectrum shown in Fig. 2. The m/z difference between b_1 _and b_2 _is equivalent to that which is between y_8 _and y_7 _as they represent the same amino acid 'Alanine', at 71.04 Dalton. Likewise, the m/z difference between b_2 _and b_3 _is equivalent to that which is between y_7 _and y_6 _as they represent the same amino acid 'Glycine', at 57.02 Dalton, and so on. This observed symmetry is a very useful feature as it can be used to determine the quality of the spectrum generated from the mass spectrometer. If a given spectrum contains all the b-ions and y-ions of a peptide, the self-convolution of the mass spectrum would be produced the highest peak when all the corresponding b-ions and y-ions peaks are aligned. For example, for the spectrum shown in Fig. 2, the highest peak would occur when y_7_, y_6_, y_5_, y_4_, y_3_, y_2 _correspond to b_2_, b_3_, b_4_, b_5_, b_6_, b_7 _are aligned on the m/z axis. This peaks occurs theoretically at the mid-point of the self-convolution results.

To verify the observation, the molecular weights of the theoretical b- and y-ions were generated for peptide sequence [**MTDQEAIQDLWQWR**], using **MS-Digest **[45].

The b-ions thus obtained are:

b = [233.10, 348.12, 476.18, 605.22, 676.26, 789.35, 917.40, 1032.43, 1145.51, 1331.59, 1459.65, 1645.73];

The y-ions generated are:

y = [1688.80, 1587.76, 1472.73, 1344.67, 1215.63, 1144.59, 1031.51, 903.45, 788.42, 675.34, 489.26, 361.20, 175.12];

A time series data is then created such that the starting mass is 0 Dalton and the ending mass is 1819.84 Dalton, which is the mono-isotopic peptide precursor mass (MH+), with an interval of 0.01 Da. The following conditions are used to set the intensity of the time series data:

data(n)={100if m/z=b(n)orm/z=y(n)else(random noise level n)0≤n≤1
 MathType@MTEF@5@5@+=feaafiart1ev1aaatCvAUfKttLearuWrP9MDH5MBPbIqV92AaeXatLxBI9gBaebbnrfifHhDYfgasaacH8akY=wiFfYdH8Gipec8Eeeu0xXdbba9frFj0=OqFfea0dXdd9vqai=hGuQ8kuc9pgc9s8qqaq=dirpe0xb9q8qiLsFr0=vr0=vr0dc8meaabaqaciaacaGaaeqabaqabeGadaaakeaacqWGKbazcqWGHbqycqWG0baDcqWGHbqycqGGOaakcqWGUbGBcqGGPaqkcqGH9aqpdaGabeqaauaabaqacqaaaaqaaiabigdaXiabicdaWiabicdaWaqaaiabdMgaPjabdAgaMjabbccaGiabd2gaTjabc+caViabdQha6jabg2da9iabdkgaIjabcIcaOiabd6gaUjabcMcaPaqaaiabd+gaVjabdkhaYbqaaiabd2gaTjabc+caViabdQha6jabg2da9iabdMha5jabcIcaOiabd6gaUjabcMcaPaqaaiabdwgaLjabdYgaSjabdohaZjabdwgaLbqaaiabcIcaOiabdkhaYjabdggaHjabd6gaUjabdsgaKjabd+gaVjabd2gaTjabbccaGiabd6gaUjabd+gaVjabdMgaPjabdohaZjabdwgaLjabbccaGiabdYgaSjabdwgaLjabdAha2jabdwgaLHqaciab=XgaSjabbccaGiab=5gaUjabcMcaPaqaaaqaaiabicdaWiabgsMiJkabd6gaUjabgsMiJkabigdaXaaaaiaawUhaaaaa@7A86@

**Figure 7:**
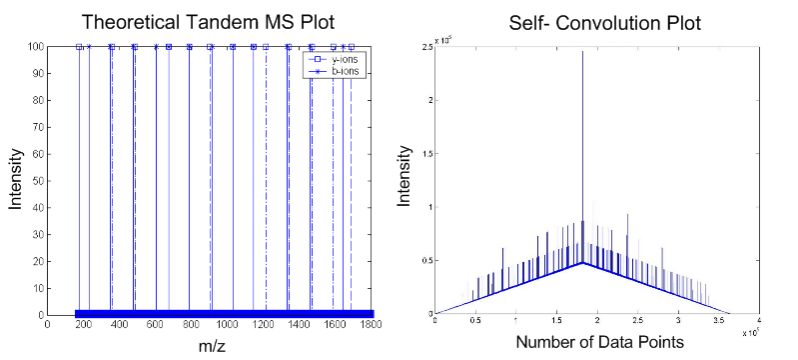
**Self-convolution plot for noise amplitude = 1**. This figure shows the result of self-convolution when noise peaks of amplitude 1 is added to the theoretical tandem MS.

A plot of these b-ions and y-ions and the self-convolution values are shown in the Fig. 7. From this figure, we observed a high peak occurs at the mid-point of the self-convolution, where the b-ions (b_n_, b_n-1_, b_n-2_, ... b_2_) align with corresponding y-ions (y_2_, y_3_, y_4_, ... y_n_). However, it is also noted that the cumulating sum of the product of all the points steadily increases from 0 to the mid-point and reducing thereof, forming a triangle below the peaks. This is potentially damaging to the detection of the peaks especially when significant noise levels are present, compounded by low intensity of b-ions and/or y-ions peaks and missing peaks, as we will demonstrate later. To determine the effects of increasing noise levels, we change the noise level to 10 as shown below.

data(n)={100if m/z=b(n)orm/z=y(n)else(random noise level n)0≤n≤10
 MathType@MTEF@5@5@+=feaafiart1ev1aaatCvAUfKttLearuWrP9MDH5MBPbIqV92AaeXatLxBI9gBaebbnrfifHhDYfgasaacH8akY=wiFfYdH8Gipec8Eeeu0xXdbba9frFj0=OqFfea0dXdd9vqai=hGuQ8kuc9pgc9s8qqaq=dirpe0xb9q8qiLsFr0=vr0=vr0dc8meaabaqaciaacaGaaeqabaqabeGadaaakeaacqWGKbazcqWGHbqycqWG0baDcqWGHbqycqGGOaakcqWGUbGBcqGGPaqkcqGH9aqpdaGabeqaauaabaqacqaaaaqaaiabigdaXiabicdaWiabicdaWaqaaiabdMgaPjabdAgaMjabbccaGiabd2gaTjabc+caViabdQha6jabg2da9iabdkgaIjabcIcaOiabd6gaUjabcMcaPaqaaiabd+gaVjabdkhaYbqaaiabd2gaTjabc+caViabdQha6jabg2da9iabdMha5jabcIcaOiabd6gaUjabcMcaPaqaaiabdwgaLjabdYgaSjabdohaZjabdwgaLbqaaiabcIcaOiabdkhaYjabdggaHjabd6gaUjabdsgaKjabd+gaVjabd2gaTjabbccaGiabd6gaUjabd+gaVjabdMgaPjabdohaZjabdwgaLjabbccaGiabdYgaSjabdwgaLjabdAha2jabdwgaLHqaciab=XgaSjabbccaGiab=5gaUjabcMcaPaqaaaqaaiabicdaWiabgsMiJkabd6gaUjabgsMiJkabigdaXiabicdaWaaaaiaawUhaaaaa@7B74@

**Figure 8:**
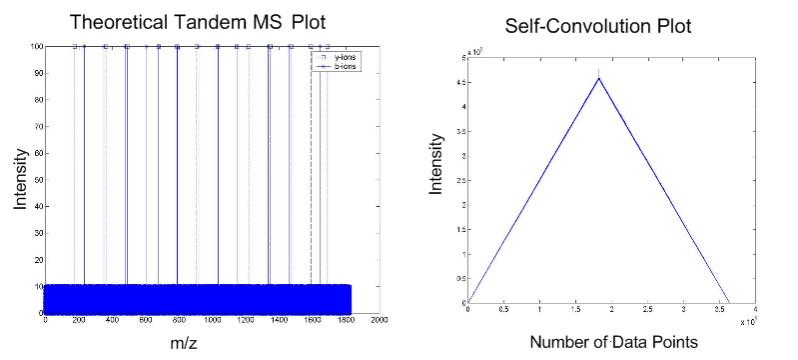
**Self-convolution plot for noise amplitude = 10**. This figure shows the result of self-convolution when noise peaks of amplitude 10 is added to the theoretical tandem MS.

We observe that, while the noise level is only 10% of the ions intensity as shown in Fig. 8, the distinctive mid-point peak is significantly reduced in comparison to the increased overall overlapping convolution values. The other observable peaks in Fig. 7 are also lost in view of the greatly increased overlapping convolution values due to augmented in noise levels. This problem can be resolved by applying convolution theorem and by removing the DC component of the product of Fourier transforms before performing the inverse Fourier transform. According to Convolution Theorem, convolution is achieved by first applying the Discrete Fourier Transform (DFT) onto the data sets, multiply these two transforms, and then perform the inverse DFT. The key point is that the near DC components are removed by setting the first 10 points of the DFT product to 0. Finally the data is normalised against its largest magnitude. The pseudo-codes are shown as below:

D = DFT(data); // compute the Discrete Fourier Transform from the spectrum

D = Df * Df; // compute the product of the DFT

DD(1:10) = 0; // remove the near-DC components from the spectrum

IDD = abs(iDFT(DD)); // compute the amplitude of the inverse Discrete

// Fourier Transform

NIDD = IDD/max(IDD); // normalised self-convolution value

**Figure 9:**
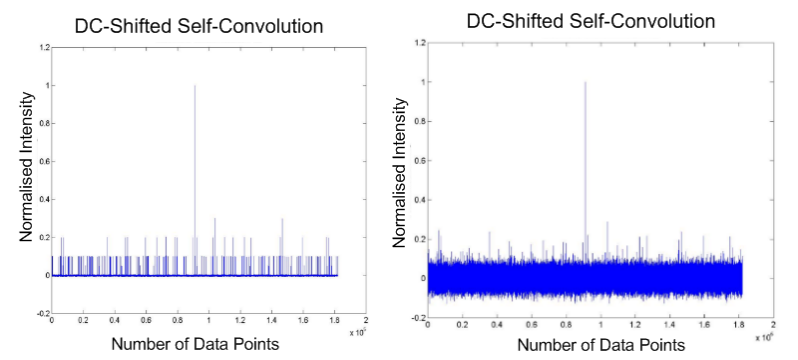
**DC-shifted self-convolution plot for noise amplitude = 1 and 10**. This figure shows the DC-shifted self-convolution results of theoretical tandem MS with noise amplitude = 1 (left) and noise amplitude = 10 (right).

As depicted in Fig. 9, we have eliminated the detrimental effects of noise by preserving the maximum peak at the mid point and the other observable peaks as compared with Fig. 8. The removal of near DC component and an additional normalization step have improved our ability to determine the quality of the spectrum.

### Quantitative measurement

We further propose a quantitative method to determine the quality of a given tandem MS spectrum from the self-convolution values, as follows:

1) Determine the maximum peak value occurs at the mid-point of the normalised self-convolution values (*P*_max(*mid *- *point*)_) within the +/- 2 Dalton error windows of the MS fragment ion mass values.

2) Find the *N *highest peaks to the left of (*P*_*L*_) and *N *highest peaks to the right of (*P*_*R*_) the mid-point peak value. The choice of *N *value ranges from 10 to 30, depending on the mono-isotopic peptide precursor mass of the fragment.

3) Calculate the ratio of the maximum mid-point peak to the average of the highest peaks to the left and right of the mid-point peak.

We term this ratio as the Quality Score (QS) of the tandem MS spectrum as shown in the following equation:

QS=Pmax⁡(mid−point⁡)12N(∑n=1NPLn+∑n=1NPRn)
 MathType@MTEF@5@5@+=feaafiart1ev1aaatCvAUfKttLearuWrP9MDH5MBPbIqV92AaeXatLxBI9gBaebbnrfifHhDYfgasaacH8akY=wiFfYdH8Gipec8Eeeu0xXdbba9frFj0=OqFfea0dXdd9vqai=hGuQ8kuc9pgc9s8qqaq=dirpe0xb9q8qiLsFr0=vr0=vr0dc8meaabaqaciaacaGaaeqabaqabeGadaaakeaacqWGrbqucqWGtbWucqGH9aqpdaWcaaqaaiabdcfaqnaaBaaaleaacyGGTbqBcqGGHbqycqGG4baEcqGGOaakcqWGTbqBcqWGPbqAcqWGKbazcqGHsislcqWGWbaCcqWGVbWBcyGGPbqAcqGGUbGBcqGG0baDcqGGPaqkaeqaaaGcbaWaaSaaaeaacqaIXaqmaeaacqaIYaGmcqWGobGtaaWaaeWaaeaadaaeWbqaaiabdcfaqnaaBaaaleaacqWGmbatdaWgaaadbaGaemOBa4gabeaaaSqabaaabaGaemOBa4Maeyypa0JaeGymaedabaGaemOta4eaniabggHiLdGccqGHRaWkdaaeWbqaaiabdcfaqnaaBaaaleaacqWGsbGudaWgaaadbaGaemOBa4gabeaaaSqabaaabaGaemOBa4Maeyypa0JaeGymaedabaGaemOta4eaniabggHiLdaakiaawIcacaGLPaaaaaaaaa@5E85@

**Figure 10:**
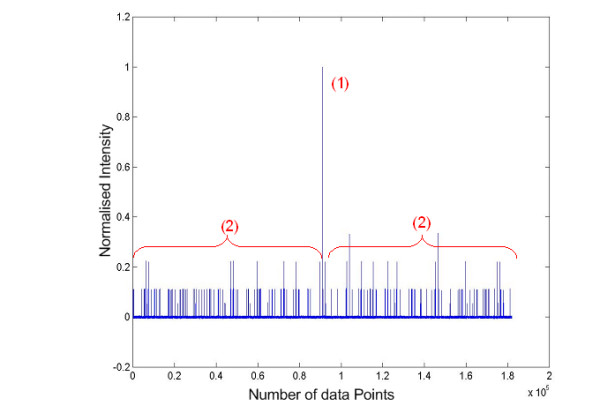
Qualitative measurement of spectrum quality.

**Figure 11:**
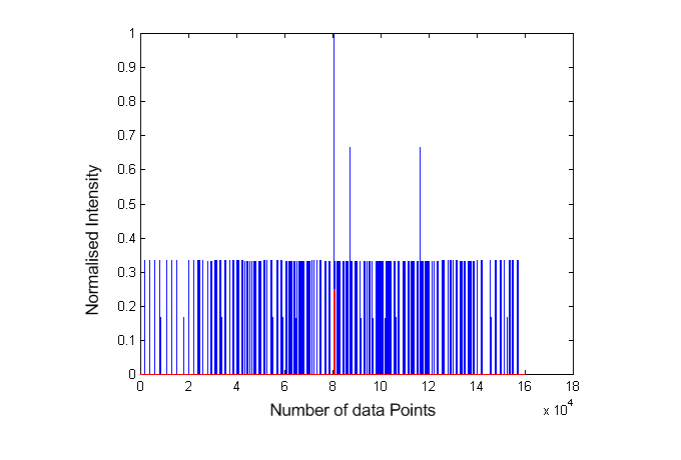
DC-shifted self-convolution of good quality mass spectrum.

Fig. 10 shows the actual components considered in our quantitative method described above. Fig. 11 shows the normalised self-convolution plot of a good tandem mass spectrum. We can see clearly that the score is higher (QS = 3.0833) in this case as compared to those shown in Fig. 4 (QS = 1.9907) and Fig. 6 (QS = 1.8030). We performed MASCOT database search to confirm the quality of these spectra.

## Availability and requirements

Project name: MS Quality Assessment

Operating system(s): UNIX or Windows

Programming language: MATLAB version 5.3, no special toolbox needed.

Licence: Email request to author.

Any restrictions to use by non-academics: Licence needed.

## Competing interests

The author(s) declares that there are no competing interests.

## Authors' contributions

CKW proposed the initial implementation of the algorithm and tested the functionality of the codes. He was involved in drafting the manuscript. LT investigated the symmetry property and helped improve the final quantitative measurement of the mass spectra. He revised the manuscript. All authors read and approved the final manuscript.
